# Key Factors Influencing Use of Immunization Cost Evidence in Country Planning and Budgeting Processes: Experiences From Indonesia, Tanzania, and Vietnam

**DOI:** 10.9745/GHSP-D-21-00264

**Published:** 2022-02-28

**Authors:** Annette Ozaltin, Kelsey Vaughan, Kassimu Tani, Fatuma Manzi, Vu Quynh Mai, Hoang Van Minh, Soewarta Kosen, Lora Shimp, Logan Brenzel, Laura Boonstoppel

**Affiliations:** a ThinkWell, Washington, DC, USA.; b Ifakara Health Institute, Dar es Salaam, Tanzania.; c Hanoi University of Public Health, Hanoi, Vietnam.; d National Institute of Health Research and Development, Jakarta, Indonesia.; e John Snow, Inc, Washington, DC, USA.; f Bill & Melinda Gates Foundation, Washington, DC, USA.

## Abstract

In many low- and middle-income countries, planning cycles and policy decisions are not always informed by cost evidence, even where relevant and recent cost evidence is available. The Immunization Costing Action Network (ICAN) project was a research and learning community designed to strengthen country capacity to generate immunization cost evidence and to understand and improve the evidence-to-policy linkages for the evidence. We identified key factors that increase the likelihood that health policy makers will use evidence for policy making or planning, which shaped the development of a 6-step evidence to policy and practice (EPP) facilitated process. ICAN used the EPP process in Indonesia, Tanzania, and Vietnam from 2016–2019. The experience resulted in several insights regarding country priorities related to cost evidence and factors that determine uptake. Cost evidence is more likely to be used if it answers a specific policy question prioritized by the immunization program, while the use case is less clear and urgent for routine planning and program management. Nonhealth ministries and subnational stakeholders can provide important perspectives to inform the research and its usability. The use case for evidence should be revisited periodically as divergences from formal planning cycles are common and new policy windows open. Ensuring evidence is available at the right time is critical, even if this requires a sacrifice between rigor and speed. Engaging a small group of stakeholders, rather than an individual, to champion the research may be more effective, and the research has greater legitimacy if it is produced by multidisciplinary country teams. Evidence and messages should be tailored for and packaged targeting different audiences. Going forward, continued support is necessary to bridge the divide between those who generate cost evidence and those who translate evidence for policy and planning decisions.

## Abstract (precis)

The evidence to policy and practice facilitated process represents a journey that countries and their development partners can embark on to increase the likelihood that health policy makers will use cost evidence for policy making and planning.

## Teaser

**Key Messages:**

- Simply making immunization cost evidence available will not likely lead to uptake at country level, with several potential causes identified.
- Facilitating the 6-step evidence to policy and practice process led to increased recognition by national- and subnational-level stakeholders of the importance of generating and using cost evidence in all 3 countries. However, this did not necessarily translate to actual use.
- Six lessons learned can help future researchers improve the use of immunization cost evidence in country planning and budgeting processes.

**Key Implications:**

- Researchers need to provide a clear use case for cost evidence. Engaging a small multidisciplinary stakeholder group to develop, implement, and later champion the research can help.
- Researchers should make cost evidence available within windows of opportunity required by policy makers and practitioners, even if it occasionally requires a sacrifice between rigor and speed. Researchers need help from local stakeholders to properly tailor, package, and deliver the key messages.
- Researchers need to be prepared to support potential users of cost evidence in translating cost data for use and should build this time into project and funding cycles.

## BACKGROUND

Governments need to understand what it costs to deliver vaccines to reach coverage goals, address health equity, manage the introduction of new vaccines, and ensure efficient use of resources. Even in low- and middle-income countries where relevant and recent cost evidence is available, planning cycles and policy decisions are not always informed by evidence. Globally, there is limited understanding of how economic or other types of evidence are used in health policy processes and decision making and how influential they are.^1^ A greater understanding of the role and use of cost evidence in these technocratic—but often deeply politicized—processes is needed.

Costing and cost-effectiveness studies have not had as significant an impact as they could have had in vaccine policy and decision-making processes where domestic and institutional politics often dominate.^2^ Even when strong evidence and decision-making frameworks exist, relationships and politics often eclipse evidence in influence.^2^ At times, rather than having the evidence inform the decision, evidence is generated to support a political decision that has already been taken, such as in the case of a new vaccine introduction.^3^^–^^5^ Further, 2 large immunization costing and financing initiatives—the EPI Costing and Financing of Routine Immunization and New Vaccines (EPIC) project^6^ and the ProVac Initiative^7^—found that although countries had developed robust cost evidence, its translation into plans and budgets was limited.^8^^,^^9^

Simply making evidence available will not lead to uptake, with several potential causes identified. First, operational costs may play a larger role than economic evidence in decision making.^10^ Supporting vaccine introduction decisions in Latin America and the Caribbean, the ProVac Initiative focused on the economic impact, whereas countries also needed information to estimate the budgetary impact of a vaccine.^7^ Second, cost evidence needs to be introduced at the right time. For several EPIC countries, the window during which costing evidence could have been introduced in the planning process did not align with project timing. Third, variation in results can be difficult for policy makers to interpret, as seen in the EPIC project countries. Fourth, immunization program managers, typically the primary customers of immunization costing research, may not actually wield as much decision-making power as is perceived to improve immunization program efficiency.^11^ In 2015, a group of global and country immunization costing and financing experts concluded that the relevance of cost data for country use needed to be strengthened, findings needed to be translated into clear messages to be useful for implementation, and the decision space of national and subnational immunization managers needed to be unpacked.^12^

Simply making evidence available will not lead to uptake, with several potential causes identified.

### Immunization Costing Action Network Project Overview

To address the identified gaps, the Immunization Costing Action Network (ICAN)^13^ project invested in strengthening the capacity of countries for economic evidence generation and evidence-based decision making and planning. From 2016–2019, ICAN worked to improve the interpretation and translation of relevant cost evidence and to understand its evidence-to-policy linkages.

ICAN was a research and learning community to increase the visibility, availability, understanding, and use of immunization delivery cost information. It was designed such that countries led the agenda, with technical facilitation and coaching from ThinkWell and John Snow, Inc. Country-based research institutions, led by local principal investigators, conducted immunization costing studies addressing challenges at the top of domestic immunization agendas, driving the research from conceptualization to dissemination, and supporting policy translation. The research institutions included a collaboration between Universitas Indonesia and the National Institute of Health Research and Development in Indonesia, the Ifakara Health Institute in Tanzania, and the Hanoi University of Public Health in Vietnam. The research institutions led country teams comprised of health economist researchers, immunization managers, and budget officers and planners from ministries of health (MOHs). The 3 country teams were convened several times during the 3-year project in workshops aimed at improving the capacity to design research, sharpen methods, and learn from each other regarding how to interpret and leverage cost evidence for policy making and planning.

## EVIDENCE TO POLICY AND PRACTICE FACILITATED PROCESS

We conducted semistructured interviews with key informants and a literature review on other initiatives and research to summarize the existing evidence around factors that influence the uptake of costing evidence. This resulted in 8 key factors that increase the likelihood that health policy makers will use evidence for policy making or planning (Box).

BOX **Key Factors That Improve Evidence Use for Policy Making or Planning**

**Demand**: Policy maker and practitioner demand for the evidence regarding their broader policy and program imperatives increases the likelihood that it is used.^16^ A country champion can help elevate its importance, thereby increasing demand.

**Right time/right place**: Evidence needs to be made available for key policy moments or within windows of opportunity required by policy makers and practitioners to act on it. Researchers need to deploy strategic opportunism within those windows or create windows to increase the likelihood of use.^17^^,^^18^

**Evidence strength and quality**: Evidence is considered more credible if it is developed following an endorsed and rigorous methodology. Analysis needs to be done by a well-respected source.

**Relationships and networks**: Evidence is more likely to be used if a focal point from within the Ministry of Health is engaged in leading or coordinating the research and if research teams are multidisciplinary with the various stakeholders involved from the beginning.^17^ Interactions between researchers and policy makers on committees and in informal relationships can also support uptake.

**Decision space**: An understanding of the decision space and types of decisions that can be affected by evidence at various levels of the health system can help ensure the relevance of the evidence for different stakeholder groups and increase its usability.

**Packaging and targeting**: Simple, attractive, easy-to-understand formats (nontechnical summaries) and clear and definitive takeaway points and requested actions are important for busy policy makers and practitioners.^1^^,^^19^

**Policy translation**: The format of the evidence might require adaptation to accommodate what is needed for policy or program use. Policy makers and practitioners may benefit from additional support to extrapolate the evidence to different contexts, questions, or geographic settings. Because researchers may not have the interest, time, or skillset for policy translation or policy advocacy, engaging policy actors can help.^19^

**Implementation climate**: Stakeholder readiness for use of the evidence can depend on their receptiveness to using economic data. Country fiscal space issues, policy priorities, and political and administrative structures, as well as the nature of institutions, can also support or impede the use of evidence.

Based on the identification of these critical success factors, we used a 6-step evidence to policy and practice (EPP) facilitated process in each country to increase the likelihood of generating policy and program-relevant cost evidence and improving its uptake and use (Figure). Supplement 1 presents the EPP facilitated process along with key questions and objectives driving each process step.

**FIGURE:**
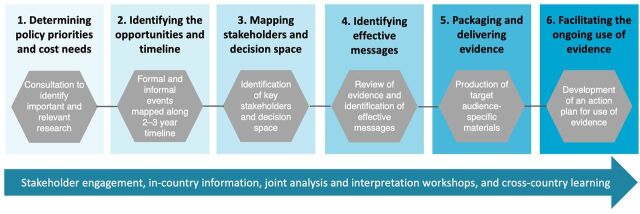
Evidence to Policy and Practice Facilitated Process

### 1. Determining Policy Priorities and Cost Needs

We aimed to identify real country demand for cost evidence. We consulted the primary customers for the research (national immunization managers and MOH budget officers) and other immunization stakeholders from government ministries, donors, and development partners to understand their high-level objectives and priorities, how cost evidence might help them achieve those objectives, and any barriers to the use of cost evidence. Through this process, country researchers became embedded in the network of immunization stakeholders and gained a strong understanding of the challenges that policy makers and program managers face. We approached stakeholders with a short menu of potential research topics, and they selected the costing of alternative vaccine delivery strategies as the most relevant. Within this topic, the priorities varied by country (Table).

We aimed to identify real country demand for cost evidence.

**TABLE.** Country Research Questions and Use of Cost Evidence

**Country**	**Research Question**	**Primary Uses of Cost Evidence**
Indonesia^21^	Using a combination of delivery strategies (health post, health center, and school), what are the district/city level costs incurred for immunization delivery that contribute to achievement of high coverage?	Allocation of the operational budget for the immunization program at central, district, and city levels in the 2 years after the study.
		Budget preparation and planning of the coronavirus disease (COVID-19) vaccination program.
		Potential future use includes advocacy with the national parliament, and development of the comprehensive multiyear plan.
Tanzania^20^	What is the average delivery cost to immunize children up to 18 months in rural and urban areas at current coverage levels and using the current mix of delivery strategies (fixed facility, outreach, and mobile)?	Development of the costing section of the national immunization strategy. Subsequent use in national and subnational planning cycles.
Vietnam^22^	What is the program cost of transitioning from tetanus toxoid vaccination of women of childbearing age to tetanus-diphtheria vaccination of children aged 7 years?	Support to the national immunization program in developing guidelines for the implementation of and budgeting for tetanus-diphtheria vaccination, including delivery strategy options.
		Guidance to provinces in preparation of their vaccination plans and budgets.
		Preparation of the immunization program’s plan for 2021–2025.

### 2. Identifying the Opportunities and Timeline

At various points during the project, we consulted with a broad set of stakeholders to identify promising opportunities for use of cost evidence. We documented the timing and approval processes for policy development and planning cycles, upcoming policy decisions (e.g., new vaccine introductions), and program management activities (e.g., preparation of the immunization comprehensive multiyear plan).

During cross-country workshops, we conducted a mapping exercise of the annual planning and budgeting process in each country. We specified the events where cost evidence could be introduced, the tactics and tools to deploy to increase the likelihood of the evidence being taken up, and any anticipated bottlenecks to the use of evidence.

### 3. Mapping Stakeholders and Decision Space

We identified key stakeholders involved in policy formulation, planning, and budgeting to determine to whom (people and institutions) the cost evidence should be targeted for greatest impact and the best ways to reach them with cost evidence. Originally, we envisioned a decision space mapping exercise involving a broad range of stakeholders to capture connections beyond costing, immunization, and even the health sector. However, we ultimately deprioritized this as we focused on generating evidence for routine policy and program activities as opposed to policy decisions that require significant advocacy and decision-making authority beyond the immunization program or health sector.

We focused on generating evidence for routine policy and program activities as opposed to policy decisions that require significant advocacy and decision-making authority.

### 4. Identifying Effective Messages

When preliminary findings became available, we came together for a cross-country analysis and evidence interpretation workshop. Country teams discussed the findings, began to formulate conclusions, posed questions to provide direction for additional analysis, identified key messages, and further delineated use cases for the cost evidence. We conducted a similar exercise with a broader group of stakeholders during dissemination activities in each country.

### 5. Packaging and Delivering Evidence

We shared best practices in presenting data to different audiences, and country teams gained confidence in developing slides and charts, writing policy briefs, and crafting an elevator pitch. We developed a dissemination plan for the results, including high-level immunization meetings at which a broad set of stakeholders would be present, followed by other smaller targeted meetings. The principal investigators also gained additional experience in presenting the findings through remote coaching sessions and through delivery of their presentation at the 2019 International Health Economics Association Congress.

### 6. Facilitating the Ongoing Use of Evidence

Alongside dissemination of the evidence, the country teams developed EPP action plans to support the uptake of the cost evidence after the exit of the researchers. Each country’s EPP plan summarized information related to each process step, clarified needed action for policy translation, and specified the technical support and resources required to ensure those actions were carried forward. For example, in Vietnam, the action plan specified a subnational dissemination plan along with information on a vaccine introduction pilot based on the results. In the case of Tanzania, the action plan included additional analyses that the national immunization program planned to commission to inform the next comprehensive multiyear plan and upcoming policy guidelines (Supplement 2).

Alongside dissemination of the evidence, the country teams developed EPP action plans to support uptake of the cost evidence after the exit of the researchers.

The EPP plans were distributed to key country stakeholders during the dissemination process. In Vietnam, the national immunization program was the owner of the action plan and ensured the next steps were carried out. In Tanzania, an EPP workshop focused on reviewing the cost results and the action plan with a broad group of government and partner stakeholders, who inputted further into the action plan and identified the resource needs required for policy translation. In Indonesia, a broad group of recipients received the action plans—including different partner organizations and units in MOH and the planning ministry—although the national immunization program did not carry the action plan forward. The Table provides an overview of the research questions and use of the cost evidence to date.

## LESSONS LEARNED

At each step during the EPP process, we validated existing global knowledge and obtained new insights. We summarize our key lessons learned regarding country priorities related to cost evidence and the factors that increase the likelihood that health policy makers will use it for policy or planning.

### 1. Provide a Clear Use Case for Uptake of Evidence

Demand for cost evidence is not enough; uptake of evidence requires a clear use case. Previous studies have emphasized the need for evidence to be relevant for policy makers to use it, and to target the needs of decision makers.^1^^,^^14^^–^^16^ Though we learned that even if there is strong demand for costing evidence from policy makers and practitioners, and policy recommendations are offered, a concrete use case must accompany the specific policy and management questions that the cost evidence is informing. In Vietnam, where the evidence was linked to a clear vaccine introduction policy decision, uptake of the evidence was more straightforward than in Tanzania and Indonesia, where evidence had been intended for routine program management and planning. Vietnam’s national immunization program quickly mobilized to pilot test a proposed change in the vaccination schedule based on the cost results from the study. The cost evidence was packaged together with burden of disease and vaccine efficacy data and presented to the National Immunization Technical Advisory Group. Due to the pressing and specific decision that the cost evidence was generated for, its use was clear and immediate.

This contrasted with the situation in Tanzania, where stakeholders identified the need for more support and further tailoring of the cost evidence to particular populations to take it forward for program management and planning. In Indonesia, government representatives thought additional data collection and analysis were needed for policy or planning relevance. As a large and diverse country, a large representative sample was cost-prohibitive. This limited the generalizability, and government stakeholders had trouble seeing a use for the results beyond the 2 provinces where the research was conducted. During the design stage, a realistic examination would have been useful to determine the likelihood that research with a small sample would be accepted to inform broader change.

### 2. Consult Nonhealth and Subnational Stakeholders to Improve Research Usability

Nonhealth ministries and subnational stakeholders can provide important insights to inform the research and its usability. As mentioned in the literature, evidence needs to be relevant to local contexts and available at a micro level.^16^ Subnational immunization managers would have added value in early consultations and throughout the project, especially for the decentralized health systems of Indonesia and Tanzania. In these countries, national-level budgets are concerned with vaccine and vaccine supply costs; whereas delivery costs (the focus of our research) are mostly met by the subnational level. In Tanzania, subnational planners fall under the ministry of local government, and engaging them in the research design would have helped ensure that the cost evidence could support their work on vaccine delivery. Similarly, including them in our country teams would have helped to build their capacity to use cost evidence for planning and to advocate for additional resources from the national level.

In addition, officers from the ministries of planning and finance who are responsible for the health budget could have provided useful insights along with higher-level policy input and buy-in. In Indonesia, for example, we did not engage the planning ministry from the beginning of the study, and therefore, we missed opportunities to include the evidence in their influential planning and budgeting process. By consulting with these ministries and even with other programs within the MOH, a discussion on how to generate evidence with potential broader relevance would have been useful. For example, how to manage shared costs and how data collection for the immunization program could benefit evidence use by other ministries or health programs. In addition, consultations with civil society organizations and other advocacy groups that typically have limited access to public cost evidence could have provided valuable insights to shape the research.

Officers from the ministries of planning and finance who are responsible for the health budget could have provided useful insights along with higher-level policy input and buy-in.

### 3. Research From Multidisciplinary Country Teams Has Greater Legitimacy

In line with findings from the literature, we found that having country teams driving the research and the focus on deeper ongoing engagement with the government resulted in research with greater policy and program relevance.^1^^,^^17^^–^^19^ Country researchers were active, not passive, players in designing, undertaking, and communicating the research findings. The research had more legitimacy from the perspectives of policy makers and practitioners due to the involvement of multidisciplinary teams (i.e., researchers and government officials) to generate and interpret evidence. However, we found that although this process benefited from having broader participation and buy-in, the process was slower.

The importance of relationships has been identified as a key factor facilitating the uptake of evidence in earlier research, which was confirmed throughout our process as well.^14^^,^^17^ The extent to which the principal investigators had already collaborated with the immunization programs on commissioned research, sat on committees together, and knew each other personally was an aid to the studies. Vietnam was a particularly successful engagement, given the research partner’s strong relationship with the national immunization program to help shepherd the EPP steps forward.

### 4. Make Evidence Available Within Windows of Opportunity

Identifying the windows of opportunity required by policy makers and practitioners to be able to act on the cost evidence is critical, even if it occasionally requires a sacrifice between rigor and speed, as recognized by other researchers.^17^^,^^18^^,^^20^^,^^21^ Key stakeholders need to be equipped with the information they demand with ample time before budget meetings. Working backward from important milestones is necessary to allow sufficient time for additional analysis and data transformations. Due to research delays, we missed an opportunity to introduce evidence into the Indonesia 2020–2024 National Medium-term Development Plan.

Due to the importance of timeliness, we learned that more rapid research might occasionally be acceptable, even if it means making some methodological sacrifices (e.g., smaller sample size and no time-motion study). Over the course of the project, we revisited the use case for the cost evidence several times and identified new opportunities even as we were finalizing the analysis. New ideas also emerged regarding the usability of the evidence as additional stakeholders were reached as part of the dissemination effort.

We learned that more rapid research might occasionally be acceptable, even if it means making some methodological sacrifices.

### 5. Tailor Evidence and Messages for Different Audiences

Earlier studies have emphasized the need for research to clearly highlight key messages, as well as the need to summarize policy recommendations.^1^^,^^14^^–^^16^^,^^22^ This was confirmed during review meetings with the national immunization managers, which offered collaboration opportunities on interpretation and message generation. The stakeholders that we consulted emphasized the importance of simple, clear, definitive takeaway points that are convenient for specific audiences to digest. Messages needed to be tailored for different audiences by the level of the health system and type of stakeholder. For example, in Tanzania, national stakeholders were most interested in the urban and rural cost differences per immunized child, regional stakeholders were interested in fixed site versus outreach variation in delivery costs, and district stakeholders were interested in cost differences between rural facilities with and without nomadic populations in their catchment area.

We also found that to tailor messages effectively, a variety of stakeholder perspectives is useful for interpreting the results and identifying policy and management implications. During our third cross-country meeting, national immunization managers and budget officers had an opportunity to review the data and analyses before the final results became available. They asked for new and different ways of presenting the data, which the researchers took onboard. For example, the MOH budget officer in Tanzania struggled to see how shared costs allocated to the immunization program could be combined with cost data in different formats from other programs. To ensure that the evidence was most useful to the immunization program and broader MOH, different cost components were classified as immunization-specific or shared depending on the budgeting process.

### 6. Anticipate a Medium- to Long-Term Horizon for Policy Translation and Evidence Use

As found in other studies, the timeliness of making research outcomes available is critical.^14^^,^^17^ Due to our capacity strengthening approach, broad stakeholder consultation process, and the rigor of the research, it took 2.5 years to develop findings and key messages for dissemination. Within our 3-year funded project, this did not leave a long runway for evidence use. The 2–3 year timeline was sufficient to meet the initial use case for the evidence, but with more time, further policy dialogue and continued transformation of evidence for use would have been possible. There are myriad opportunities for use of evidence that crop up continually, and a longer time horizon allows for bigger thinking and more creativity regarding the use of the findings. One-off costing studies are expensive efforts, and the utilization of the generated evidence should be maximized. Projects that take an explicit capacity-strengthening approach should consider a longer timeline of 4–5 years to allow for this.

There are myriad opportunities for use of evidence that crop up continually, and a longer time horizon allows for bigger thinking and more creativity regarding the use of the findings.

Moreover, cost evidence for routine planning and program management should not only be produced and used once, but rather, should be used iteratively with adjustments reevaluated and streamlined over several budget cycles. Ideally, one-off costing studies should be followed and complemented by the continuous collection of costing data, such as through National Health Accounts exercises.

### 7. Strengthen Capacity for Interpreting Cost Evidence and Policy Translation

Cost evidence often may not be used due to a lack of understanding on how to transform it to answer policy or program questions. Policy translation and planning often require additional analyses and transformations to cost findings to be able to answer specific questions and be usable for budget requests. Policy translation and planning often draw on a different skillset than that of the researchers that generate the evidence, and immunization program managers and MOH budget officers have competing priorities and do not always have the right skills for this either. For example, at the national level, immunization program teams would benefit from increased capacity to use cost evidence for scenario planning, modeling, and projections. At the subnational level, managers would benefit from support in interpreting cost evidence and transforming it to their local context (e.g., matching it to local budget lines, scaling unit costs to their catchment population, and factoring local coverage and delivery strategy mix). Supplement 3 provides common examples of transformations that may be required to facilitate the use of cost evidence.

Previous research has found that the bridge between researchers and policy makers is a key barrier to use of evidence.^16^^,^^17^ Capacity strengthening of researchers is most effective when skills and confidence are built, when it supports linkages and partnerships, and when the research is “close to practice.”^15^ We found that country teams needed significant support in the presentation of findings, translating the content into plainer language, and probing on the messages and conclusions related to specific policy and program questions. In addition to focusing on interpretation and use of the evidence, ThinkWell and John Snow, Inc.’s capacity-strengthening efforts used cross-country learning, training, remote coaching, and supportive supervision to develop the country teams’ evidence generation capacity. The technical facilitators were involved at every step of the research process from the design of the study and protocol development to developing data collection tools and conducting rigorous analysis. Junior researchers were also included on the country teams to ensure that capacity of a younger generation was being built to take those skillsets forward.

Nevertheless, even more capacity strengthening to support policy translation is needed for researchers, including practicing presentation skills to deliver results and key messages. Building in extra time to support capacity strengthening in health economics was also necessary for immunization program managers and MOH budget officers. In response to their demand, the project team offered a health economics introductory workshop, but more capacity enhancement in health economics was needed. When the results were becoming available, government officials expressed a desire to have been included in the data collection effort and to gain expertise in policy and budget advocacy. To support policy translation in Tanzania, the immunization program developed a wish list for additional support which included technical assistance in developing briefers and advocacy messages for use with nonfinance stakeholders and support from health financing technical advisors to ensure use of the evidence.

Although the impact of decision space on improving the delivery of primary health care services such as immunization is still being explored, the positive relationship between decision space and institutional capacity and accountability is largely recognized.^23^^,^^24^ Our capacity-strengthening efforts were limited in that they were focused on the country research teams that carried out the work and not the institutions. Although this was beyond the scope of our project, institutional capacity for evidence-based decision making needs to be strengthened, and decision support systems in which economic evidence is considered need to be built out.^19^ Bringing in external support could be considered, for example through a consultant or embedded staff, to help strengthen the capacity of policy makers and practitioners to review economic evidence and support its ongoing use. However, this would be an interim solution, and capacity-strengthening needs to go beyond individuals, focusing on institutional capacity, although there is often less appetite for this.^22^ Moreover, beyond government institutions, demand for and use of cost evidence needs to be increased for a broader set of stakeholders—including civil society organizations and others in the immunization decision space—to improve transparency in planning and decision making. The latter requires a longer-term horizon and far more resources, political will, and regulatory support from policy makers.^22^^,^^25^^,^^26^

### 8. Form a Multistakeholder Group to Champion the Research

Previous research has shown that personal contact and relationships are important in improving the uptake of evidence.^14^^,^^15^^,^^21^ Our decision to prioritize the immunization manager as the primary champion for the research had mixed results. First, the position is an appointed one and is subject to political upheavals. Over the course of our engagement, there were 2 turnovers in immunization program managers in Indonesia. All 3 managers were supportive of our study, but while the first had selected the research question and contributed to study design, the following 2 did not have the same ownership. Second, immunization managers typically bring more of an epidemiological skillset to their position, and there was variation across the 3 countries in their level of comfort with economics and imagination on how cost evidence could improve the management of the immunization program. Third, planning and policy processes require a broad group of stakeholders, and as such, the immunization manager holds only one piece of the puzzle.

A small multistakeholder group comprised of government officials (including from nonhealth ministries), donors, development partners, and a civil society organization representative may have been more successful in guiding and championing the research and in facilitating dissemination and use. In Indonesia, the project concluded during a leadership change at the MOH when future priorities were uncertain. There was no clear champion within government to take the work forward, which a multistakeholder research steering committee may have protected against.

## CONCLUSION

Simply making data and evidence available does not result in its effective use. The EPP facilitated process represents a journey that countries and their development partners can embark on to increase the likelihood that health policy makers will use cost evidence. Although each country’s journey is unique, in all 3 project countries, the EPP process led to increased recognition of the importance of using cost evidence—an outcome that will most likely be sustained. At the beginning of the EPP process, country stakeholders intuitively knew that having cost evidence was better than not, but some had trouble articulating how it could be used. Because country stakeholders were involved throughout the EPP process and because messages were tailored to various audiences, the value of the generated cost evidence became clearer.

Although each country’s journey is unique, in all 3 project countries, the EPP process led to increased recognition of the importance of using cost evidence—an outcome that will most likely be sustained.

Nevertheless, costing to inform routine planning and budgeting is often still considered by in-country stakeholders as a “nice to have” not a “need to have.” To ensure that costing and economic evidence will continue to be used in routine management and planning processes, additional capacity strengthening is needed to increase not just the capacity to generate evidence but also increase decision makers’ capacity to interpret and use evidence. Research projects need to build in more support to bridge the divide between those who typically generate cost evidence and those that translate evidence for policy decisions and planning. Going further, we recommend building policy dialogue and policy advocacy work more explicitly into the EPP process.

Just as has been the limitation of other studies focused on improving the evidence base around EPP,^27^ it has been challenging to measure and quantify our results. Our lessons learned are largely descriptive, and future research would benefit from more explicit measurement of the 8 key factors that we have identified that increase the likelihood of evidence uptake, as well as the impact of the EPP facilitated process. A better understanding of the processes and politics around policy decisions and planning may increase the likelihood that they are more evidence-informed, if not evidence-based.

## Supplementary Material

21-00264-Boonstoppel-Supplements-1-3.pdf

## Notes

**Peer Reviewed**

**First published online:** January 25, 2022.
