# Supplementary material for: Key Factors Influencing Use of Immunization Cost Evidence in Country Planning and Budgeting Processes: Experiences From Indonesia, Tanzania, and Vietnam
Source: Glob Health Sci Pract. 2022 Feb 28;10(1):e2100264. doi: 10.9745/GHSP-D-21-00264 (PMC8885341; doi:10.9745/GHSP-D-21-00264)
Supplement: 21-00264-Boonstoppel-Supplements-1-3.pdf [file 21-00264-Boonstoppel-Supplements-1-3.pdf]

Supplement to: Ozaltin A, Vaughn K, Tani K, et al. Key factors influencing use of immunization cost evidence in country planning and budgeting processes: experiences from Indonesia, Tanzania, and Vietnam. *Glob Health Sci Pract*. 2022;10(1):2100264. https://doi.org/10.9745/GHSP-D-21-00264

SUPPLEMENT 1. IMMUNIZATION COSTING ACTION NETWORK (ICAN) EVIDENCE TO POLICY AND PRACTICE FACILITATED PROCESS

We employed a six-step Evidence to Policy and Practice (EPP) facilitated process in each country to increase the likelihood of generating policy and program-relevant cost evidence and improving its uptake and use. The word 'practice' was added to the commonly used 'Evidence to Policy' term to emphasize the importance of generating evidence not just for policy decisions, but also for use in everyday program management and routine processes such as planning and budgeting.

Step	Key Questions Underpinning Each Process Step
**1. Policy Priorities and Costing Needs**	This process step focused on generating the "right questions" that were owned by country stakeholders to drive a process of more program and policy-relevant research. Questions considered for this step included:<br>What are the top issues that are keeping immunization managers and planners up at night?<br>How can cost evidence help to support policy decisions, program management, and planning?<br>Which policy or program questions can be addressed by cost evidence in the next 2-3 years?<br>Are key stakeholders receptive to using cost evidence?
**2. Timeline and Opportunities for the Use of ICAN Evidence**	This process step comprised stakeholder consultations to identify the promising opportunities for use of cost evidence, followed by a mapping exercise of key inputs and timing for these opportunities. Questions considered for this step included:<br>Which upcoming opportunities (decisions, program management activities, routine processes) could benefit from cost evidence generated by the study?<br>What is the 2-3 year timeline for these decisions, activities, or routine processes?<br>At which points could cost evidence be introduced?<br>What is the likelihood of influencing these decisions, activities, or routine processes with cost evidence?<br>What is the implementation climate and readiness for change?
**3. Key Stakeholders and Their Decision Space**	This process step focused on identifying the people/bodies to target and the best ways to reach them with the cost evidence. Questions considered for this step included:<br>Who is involved in policy formulation, planning and budgeting, and program design?<br>Who are the influencers and decision makers?<br>What are the power dynamics between stakeholders?<br>What are the formal vs informal decision-making processes?

Step	Key Questions Underpinning Each Process Step
**4. Effective ICAN Messages and Evidence**	This process step included a joint analysis and interpretation workshop between researchers and policymakers to discuss the preliminary findings and begin to piece together the story that the results told. Questions considered for this step included:<br>Which conclusions can we draw from the cost evidence?<br>What policy or program questions can we answer with the cost evidence?<br>What are the findings that will resonate with different stakeholder groups?<br>What are the key messages that will be effective with these stakeholders?<br>What further questions do the results raise?
**5. Presenting ICAN Evidence and Messages Effectively**	This process step included preparation of different formats for presentation of the evidence and development and execution of a dissemination plan. Questions considered for this step included:<br>How should results be packaged and delivered to target audiences?<br>Which formats resonate with different audiences?
**6. Facilitating the Use of the ICAN Evidence**	This process step focused on developing a plan to facilitate ongoing use of the evidence, including elaborating the supplemental analyses required to transform the evidence for policy and program use and identifying additional technical support and resources needed. Questions considered for this step included:<br>What are the next steps in dissemination, communication, and advocacy?<br>Who is going to be responsible for carrying out the action plan to facilitate the use of cost evidence in the identified opportunities (i.e., decisions, program management activities, routine processes)?<br>What additional data or analysis is needed to transform the evidence for policy and program use?<br>What additional technical support and resources are required?<br>What are potential barriers to using cost evidence and what are tactics to overcome them?

Supplement to: Ozaltin A, Vaughn K, Tani K, et al. Key factors influencing use of immunization cost evidence in country planning and budgeting processes: experiences from Indonesia, Tanzania, and Vietnam. *Glob Health Sci Pract*. 2022;10(1):2100264. https://doi.org/10.9745/GHSP-D-21-00264

SUPPLEMENT 2. TANZANIA EPP PLAN: TRANSLATION OF EVIDENCE FOR KEY STAKEHOLDERS

Stakeholder	Priority Evidence to be Presented	Additional Analyses to Build on the Findings
National-level decision maker	The cost of a fully immunized child in rural health facilities compared to urban facilities<br>Direct and indirect costs of immunization delivery (actual costs for achieving current coverage)	Direct and indirect costs of immunization classified in line with national budget categories<br>Estimation of costs to incrementally and sustainably increase coverage)<br>The cost of a fully immunized child by income quartile
National-level partner	Immunization delivery costs are lower in rural settings when compared to urban health facilities	Additional contributions to delivery costs (including related supplies and labor) to drive advocacy for resource allocation for immunization services<br>Guidance to help different health system levels define their 'delivery package'<br>Identification of priority areas in the next year's annual planning through dissemination of findings with key stakeholders at the regional and local level<br>Define inputs into the next comprehensive multiyear plan and 5-year projections for immunization program
Regional-level decision maker	Methods of outreach delivery can have a lower cost than facility-based delivery	Costing for specific strategies (e.g., mobile services in urban areas, matrix for outreach services in nomadic areas, temporary vaccination posts, private sector engagement such as cold chain use)<br>Recommendations for specific cost reduction and delivery strategies in urban vs. rural settings<br>Review of cost and use of Reaching Every Child strategy<br>Estimation of cost for outreach vs fixed vaccination services using different scenarios<br>(e.g., marketplace sites vis-à-vis mobile in nomad/lake or mobile in urban)

Regional level decision maker	Methods of outreach delivery can have a lower cost than facility-based delivery	Costing for specific strategies (e.g., mobile services in urban areas, matrix for outreach services in nomadic areas, temporary vaccination posts, private sector engagement such as cold chain use)<br>Recommendations for specific cost reduction and delivery strategies in urban vs. rural settings<br>Review of cost and use of Reaching Every Child strategy<br>Estimation of cost for outreach vs fixed vaccination services using different scenarios<br>(e.g., marketplace sites vis-à-vis mobile in nomad/lake or mobile in urban)
District-level key decision maker	Differences in delivery costs between rural facilities with nomadic populations and facilities without nomadic populations	Indication of cost difference by presenting utility costs as associated with facility-based delivery between nomadic and non-nomadic populations

Supplement to: Ozaltin A, Vaughn K, Tani K, et al. Key factors influencing use of immunization cost evidence in country planning and budgeting processes: experiences from Indonesia, Tanzania, and Vietnam. *Glob Health Sci Pract*. 2022;10(1):2100264. https://doi.org/10.9745/GHSP-D-21-00264

SUPPLEMENT 3. COMMON EXAMPLES OF DATA TRANSFORMATIONS REQUIRED FOR POLICY DECISIONS AND PLANNING

Some transformation often has to be done to make cost findings (e.g., cost per dose by delivery strategy, cost per fully immunized child, cost by level of the health system, cost profile at health facilities) usable for budget requests or planning and policy decisions. Transformation may be required so that evidence is:

Extrapolated to different geographic settings or contextualized to local contexts, particularly if the sample size is limited.

Combined appropriately with other evidence (e.g., coverage data).

Massaged to fit with cost categories and line items in budgets, planning documents, or funding applications, such as a comprehensive multiyear plan where there might not be a 1:1 crosswalk.

Adjusted to reflect recent (or planned) expansions of the immunization program that occur after the costing year. Historical (retrospective) cost evidence captures inefficiencies and quality issues built into the routine system at the time that data were collected, requiring estimates to the cost to change the program.

Adapted for discussions on broader health sector planning and program integration or adjusted for relative comparisons to other program areas.

Used in models to estimate financial cost implications and budget impacts of various scenarios for scaling up coverage, increasing equity, or improving quality.
